# Monitoring metrics over time: Why clinical trialists need to systematically collect site performance metrics

**DOI:** 10.1177/26320843221147855

**Published:** 2022-12-21

**Authors:** Victoria Yorke-Edwards, Carlos Diaz-Montana, Macey L Murray, Matthew R Sydes, Sharon B Love

**Affiliations:** 1 MRC Clinical Trials Unit at UCL, Institute of Clinical Trials and Methodology, 4919 University College London, London, UK; 2 591739 Health Data Research UK, London, UK; 3 NHS DigiTrials, Data Services Directorate, NHS Digital, Leeds, UK; 4 British Heart Foundation Data Science Centre, Health Data Research UK, London, UK

**Keywords:** Risk-based monitoring, clinical trials, central monitoring, centralised monitoring, Study-Within-A-Trial (SWAT)

## Abstract

**Background:** Over the last decade, there has been an increasing interest in risk-based monitoring (RBM) in clinical trials, resulting in a number of guidelines from regulators and its inclusion in ICH GCP. However, there is a lack of detail on how to approach RBM from a practical perspective, and insufficient understanding of best practice.

**Purpose:** We present a method for clinical trials units to track their metrics within clinical trials using descriptive statistics and visualisations.

**Research Design:** We suggest descriptive statistics and visualisations within a SWAT methodology.

**Study Sample:** We illustrate this method using the metrics from TEMPER, a monitoring study carried out in three trials at the MRC Clinical Trials Unit at UCL.

**Data Collection:** The data collection for TEMPER is described in *DOI: 10.1177/1740774518793379*.

**Results:** We show the results and discuss a protocol for a Study-Within-A-Trial (SWAT 167) for those wishing to use the method.

**Conclusions:** The potential benefits metric tracking brings to clinical trials include enhanced assessment of sites for potential corrective action, improved evaluation and contextualisation of the influence of metrics and their thresholds, and the establishment of best practice in RBM. The standardisation of the collection of such monitoring data would benefit both individual trials and the clinical trials community.

## Background

Over the last decade there has been an increasing interest in risk-based monitoring (RBM) in clinical trials, with the FDA and EMA encouraging the use of risk-based monitoring in guidelines published in 2013.^1,2^ This attitude was then supported by the International Council for Harmonisation of Technical Requirements for Pharmaceuticals for Human Use (ICH) GCP E6(R2) guidance in December 2016.^3^ These documents broadly defined risk-based monitoring as a monitoring plan that is “tailored to the specific human subject protection and data integrity risks of the trial” and might include “a mix of centralized and on-site monitoring practices”.^2, p.10,11^

Risk-based monitoring necessitates the use of risk assessments, carried out during trial set-up and updated throughout the trial. In these risk assessments, appropriate monitoring methods are chosen to mitigate the identified risks. These methods may include central monitoring, which is performed away from the investigator research site, and usually involves the timely evaluation of accumulating data (or lack thereof) held in the trial database.^4^ Central monitoring may form a key part of the monitoring of a trial with specific metrics being chosen to monitor the integrity of the trial data and the safety of the participants. In some cases, trialists use pre-determined thresholds for these metrics to determine whether action should be taken (or ‘triggered’). Such action might be to contact the research site to discuss the finding and any necessary mitigations, with further escalation to an on-site visit if deemed more serious. In some trials, on-site visits will be an integral part of risk-based monitoring, with scheduled site visits at key points in the trial; in others, site visits may be used only under specific circumstances determined by central monitoring. Risk-based monitoring therefore seeks to choose the best monitoring methods for the identified level of risk, and the methods used may differ markedly between trials.

While risk-based monitoring has been defined, and is being encouraged in the guidelines published by the regulators, there is a lack of detail on how to practically implement RBM. This is attributable to the limited research to date showing the ‘best’ approach in RBM.^5–7^ Since the FDA and EMA guidelines of 2013 a number of Studies Within a Trial (SWATs)^8^ have reported, including ADAMON, the MONITORING study, OPTIMON, the START monitoring sub-study and TEMPER.^9–13^ However, these primarily sought to evaluate the effectiveness and economics of risk-based monitoring and central monitoring as compared to the traditional approaches of on-site monitoring and/or Source Data Verification (SDV), rather than to determine best RBM practice. A similar emphasis is found in numerous retrospective studies.^14–17^

Metrics are numeric measurements, in this case mostly obtained and calculated from data held in the trial’s database, and used to evaluate a site’s risk or performance. These metrics may be compared between sites or with set thresholds to highlight potential or actual risks and under-performance, and this may trigger an action, ranging from simply contacting the site to discuss the reasons for any issue, to conducting an on-site visit, or discussion with trial oversight committees. Academic and industry-based groups have suggested metrics that might be used in this way.^18–20^ TransCelerate suggested “risk indicators” from a wide range of categories covering safety, data quality and on-site workload.^19^ This vision of a comprehensive set of metrics contrasts strongly with a vision of a core set that could be used by all multicentre trials, proposed by Whitham et al.^20^ They used a Delphi Process to choose a set of eight key performance metrics from a large set of performance metrics identified in a systematic literature review of studies that proposed or used metrics for monitoring or measuring performance.^21^

These suggested metrics have not been tested systematically for monitoring effectiveness. Whitham et al. concluded that future research should evaluate the effectiveness of using their core metrics,^20^ and TransCelerate only called on industry partners to volunteer what had worked or not worked, rating metric changes over time as “better”, “worse” or “about the same”.^22^ This lack of systematically reported real-world experience raises important questions, such as those given in Box 1.

Box 1: Examples of research questions about the use of metrics in monitoring

• Are the suggested metrics effective for monitoring and/or comparing site performance?
• Are the suggested metrics practical or easy to use?
• Is a small set of core metrics predictive of overall site performance, or is a larger range of metrics better?
• How are thresholds best determined and managed over the course of a trial?
• How frequently should metrics be assessed?
• How should trialists use metrics to determine actions?

As a step towards answering these questions, in this paper we look to provide a method of looking at a group of metrics across time to start to understand how metrics change and are affected by trial activities. We detail how clinical trials units can track metrics and thresholds using descriptive statistics and visualisations, and show how this method has been used to further investigate data from TEMPER.^13^ We also discuss our registered SWAT protocol for those implementing our method and discuss the potential benefits from tracking and standardisation.

## Case study

### Background

This case study uses data from TEMPER.^13^ The methods are summarised in the SWAT 167 protocol published in the SWAT Store Repository.^23^ TEMPER was a study that assessed the ability of triggered monitoring to distinguish sites with important protocol or GCP compliance issues.^13^ It was run in three Phase III randomised multicentre oncology trials between 2013 and 2016. It used a prospective matched-pair design, in which findings from on-site monitoring visits of sites with a high number of metrics that breached thresholds, called ‘triggers’, were compared with those of matched sites with a low number of triggers. Each trial had separate metrics, thresholds, and review frequencies based on the trial’s own Risk Assessment and Monitoring Plan. A bespoke monitoring management system generated trigger data from data extracted from the trial databases, and produced reports to facilitate the selection of sites for on-site monitoring visits.^24^ This dataset contains a rich source of information on the behaviour of metrics against their thresholds, allowing description and visualisation of monitoring data.

### Methods

The TEMPER study was carried out by comparing paired sites, with each pair containing a site whose on-site visit was triggered by a high total trigger score or specific concerns (a ‘triggered’ visit), and a site that was matched to it, but had a low total trigger score and no particular concerns (an ‘untriggered’ visit). ‘Total trigger scores’ are the total number of metrics whose thresholds were violated at a particular site and time-point. Sites were paired once a ‘triggered’ site had been identified and they were matched if they had similar numbers of patients, and if a similar length of time had passed since their first patient had been randomised. Where there were multiple low scored candidates for matching, the site with the lowest trigger score was chosen.^13^ The original study focussed on comparing any differences in the findings between triggered and untriggered site visits. This case study focusses instead on investigating the activity of the central monitoring metrics. We looked at metrics across time to investigate:

• The behaviour of total trigger scores after a site visit
• Whether any change in the total trigger score was sustained over the following year
• Whether some triggers (indicators of whether a metric has violated a set threshold) were more sensitive.^25^

**Figure 1.**
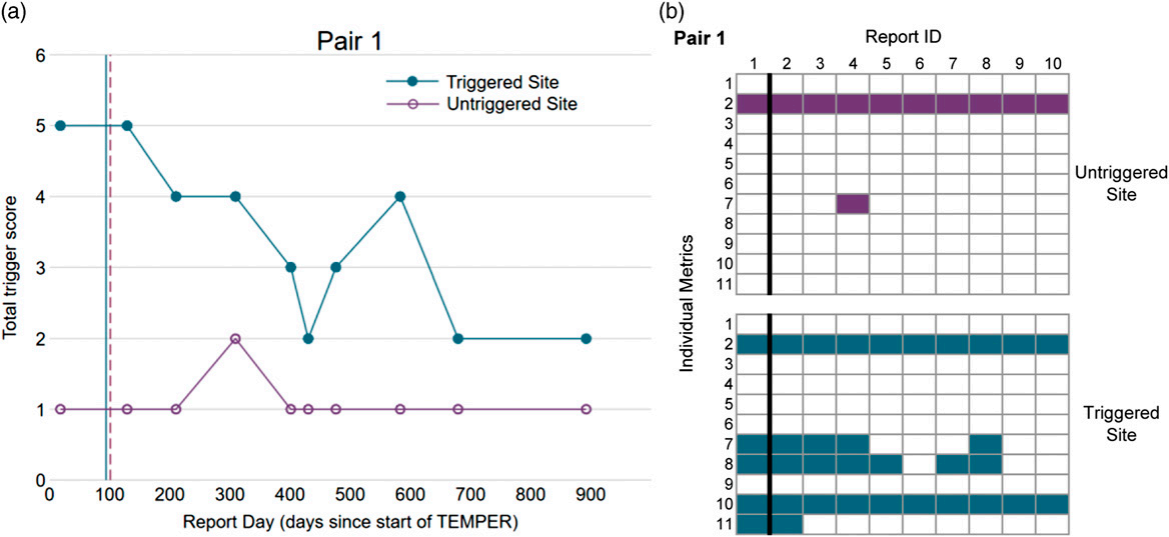
(a) Example of how trigger scores were plotted against the data extraction day [Triggered site visits shown with a solid vertical line; Untriggered site visits with a dashed line]. (b) Example of how individual triggered metrics were plotted for each sequential report in a matrix [thick vertical lines show timing of site visits].

The central monitoring metrics are visualised using Stata 15.1 and Microsoft Excel (see dataset details in Supplementary Appendix 1). Figure 1(a) presents an example. First the total trigger score is calculated for each monitoring report (indicated by a dot on the line) and plotted against the data extraction day. Separate lines are given for each of the sites in each pair, with the high trigger site marked as Triggered, and the low trigger site as Untriggered. Each on-site visit is indicated by a vertical line.

Visualising each pair in this way allows clinical trialists to see immediately the trigger activity for each site before and after any site visit, with the timing of each report shown by the circles on each plotline. However, this only shows the overall trend of the composite total trigger score.

The second visualisation (Figure 1(b)) plots each trigger of the composite over time, with cells coloured indicating when a trigger fired. Separate matrices are given for each of the sites in each pair, with the high trigger site marked as Triggered, and the low trigger site as Untriggered. Visualising the triggers in such matrices allows trialists to see the contribution of each trigger to the total trigger score. This is useful in meetings where site performance will be discussed, and to see how individual triggers have behaved across a number of reports. A line in each matrix indicates between which two reports an on-site visit took place to demark the trigger activity before and after the site visit.

Descriptive statistics were also calculated to investigate whether there was an improvement in the total trigger scores after site visits, and whether any improvement was sustained. We calculated the number of sites where the total trigger score lowered between the pre- and post- on-site visit report separately for triggered and untriggered sites, because untriggered sites may have insufficiently high total trigger scores to allow for a decline in score. We also calculated the number of sites where the post-visit trigger score had remained the same or declined further at 1 year after the visit. As with the TEMPER results paper, the trials are not disclosed.

### Results

#### Trial 1

**Figure 2.**
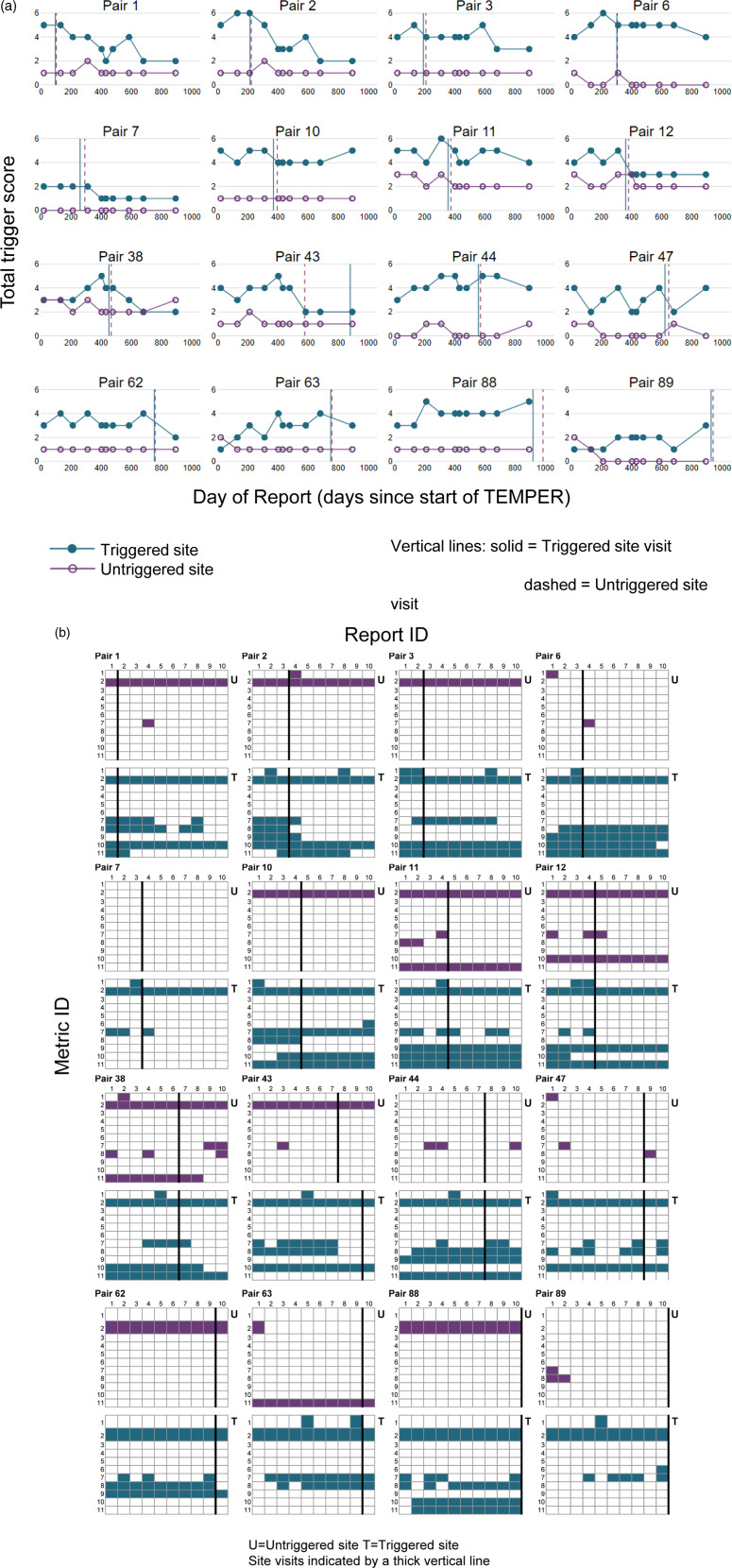
(a) Trial 1: Graphs of the total trigger score across time for triggered and untriggered sites (b) Trial 1: Matrices showing individual triggers firing.

In Trial 1, 16 pairs underwent on-site visits during its 3 years in TEMPER. Table 1 shows 14 of these pairs had both pre- and post-visit reports that could be compared; two pairs had monitoring visits at the end of TEMPER, so the visit’s effect could not be measured. 64% (9/14) of the sites that had triggered on-site visits saw a lowering of their total trigger scores from pre- to post-visit report. By comparison, 7% (1/14) of sites that had untriggered site visits saw a decrease in total trigger score. As Figure 2(a) shows, this is partly because many untriggered sites already had total trigger scores of 0 or 1. Nine pairs had post-visit reports and reports for 1 year after. 78% (7/9) of sites that had triggered on-site visits, and 89% (8/9) of those that had untriggered visits had trigger scores that remained the same or declined further a year after the visit.

**Table 1.** Trigger score activity in the TEMPER trials. Number of sites whose trigger scores decreased in the first central-monitoring report post-visit, and number of sites who score remained the same or lower 1 year after that post-visit central monitoring report.

	Trial 1		Trial 2		Trial 3	
	Triggered	Untriggered	Triggered	Untriggered	Triggered	Untriggered
	(*n* = 16)	(*n* = 16)	(*n* = 12)	(*n* = 12)	(*n* = 11)	(*n* = 11)
Trigger score lowers from pre- to post- visit	64% (9/14*)	7% (1/14*)	67% (8/12*)	0% (0/12*)	56% (5/9*)	13% (1/8*)
Trigger score same or lower from post- visit to 1 year later	78% (7/9**)	89% (8/9**)	50% (3/6**)	83% (5/6**)	100% (3/3*)	100% (5/5*)

*Sites with total trigger scores before and after the on-site visit **Sites with total trigger scores just after the site visit, and 1 year later.

Figure 2(b) reveals that several metrics were commonly triggered/untriggered for most pairs. Metric 2 remained triggered throughout TEMPER for all of the triggered sites and 10 of the 16 untriggered sites. Metrics 3, 4 and 5 were never triggered (Supplementary Appendix 2 defines all metrics in the three trials). The threshold for Metric 6 was adjusted partway through TEMPER and this may have affected the total trigger score.

#### Trial 2

**Figure 3.**
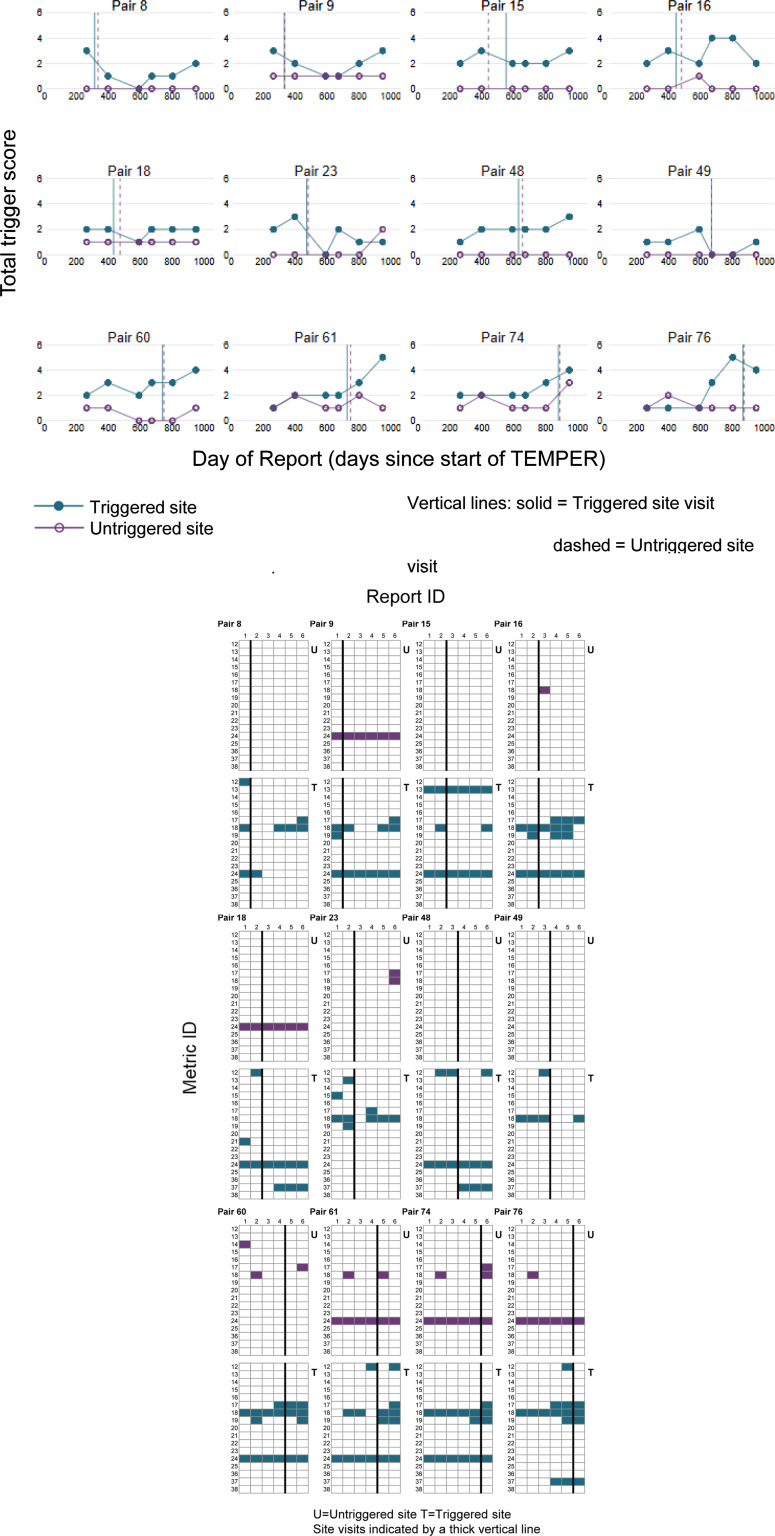
(a) Trial 2: Graphs of the total trigger score across time for triggered and untriggered sites (b) Trial 2: Matrices showing individual triggers firing.

In Trial 2, 12 pairs underwent site visits over ∼2 years as part of TEMPER. Figure 3(a) shows graphs of each pair and Figure 3(b) shows the paired matrices. Table 1 shows the descriptive statistics for this trial. All 12 pairs had both pre- and post-visit reports that could be compared. 67% (8/12) of the sites that had triggered on-site visits saw a lowering of their total trigger scores from pre- to post-visit report. None of the sites that had untriggered site visits (12) saw a decrease in total trigger score. Figure 3(a) shows this is partly because many untriggered sites already had total trigger scores of 0 or 1. However, the graphs also show that in several cases trigger scores actually rose. Six pairs had post-visit reports and reports for 1 year after. 50% (3/6) of sites that had triggered on-site visits, and 83% (5/6) of those that had untriggered visits had trigger scores that remained the same or declined further a year after the visit.

Figure 3(b) shows that while none of the metrics were constantly triggered for all sites; metric 24 was constantly triggered at 13 of the 24 sites. Seven metrics (16, 20, 22, 23, 25, 36 and 38- see Supplementary Appendix 2) were never triggered, although 16 (“Sites who have recruited more patients than a set target”), was deactivated after the trial was closed to randomisation, and metrics 36 and 38 were added partway through.^24^ Two metrics comprised the majority of the trial’s total trigger scores: metrics 18 and 24. Thresholds for metrics 17 and 19 were adjusted during the study, which may have affected whether they were triggered.

#### Trial 3

**Figure 4.**
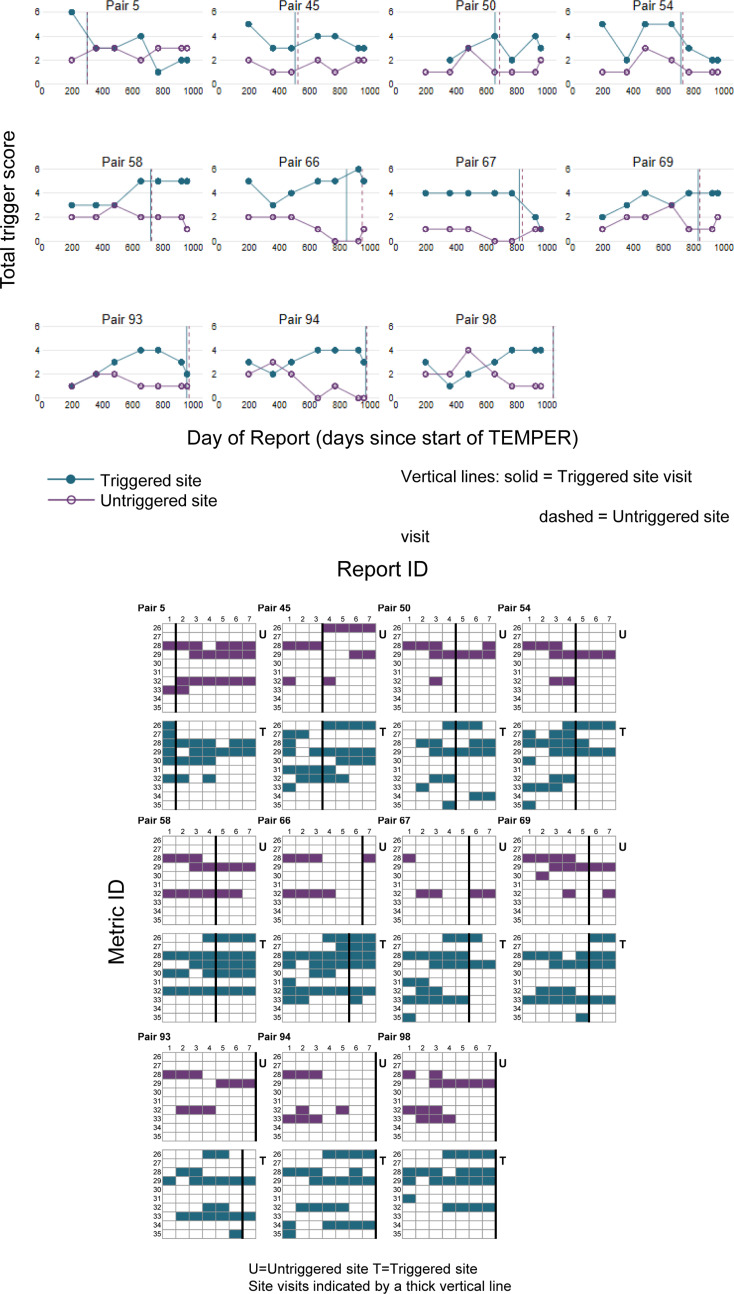
(a) Trial 3: Graphs of the total trigger score across time for triggered and untriggered sites (b) Trial 3: Matrices showing individual triggers firing.

In Trial 3, 11 pairs underwent site visits over ∼2 years as part of TEMPER. Figure 4(a) shows graphs of each pair and Figure 4(b) shows the paired matrices. 77% (17/22) of had pre- and post-visit reports (Table 1).

56% (5/9) of sites that had triggered on-site visits saw a lowering of their total trigger scores from pre- to post-visit. 13% (1/8) of sites that had untriggered site visits saw a decrease in total trigger score. As the graphs in Figure 4(a) show, this is partly because many of these untriggered sites already had total trigger scores of 0 or 1. However, the graphs also show that in several cases trigger scores actually rose. Three triggered and five untriggered sites had post-visit reports, for 1 year after. All of these had trigger scores that remained the same or declined further a year after the visit.

Figure 4 shows that the individual and total trigger scores were volatile. In three elements of Figure 4(a) the total trigger score in certain reports was higher in the untriggered site than the triggered site. Figure 4(b) shows that no metrics were constantly triggered for all or the majority of pairs, and that all metrics were triggered at least once.

## Discussion

We present a novel way to explore and understand the performance of monitoring metrics, thresholds and triggers over time, as they are the fundamental underpinning of Risk-Based Monitoring. We feel this is a useful tool and encourage others to follow. As an example, these visualisations allow us to see that the data from each of the TEMPER trials tells a different story. Table 1 indicates that in Trial 1 total trigger scores largely improved after on-site visits, and remained improved for at least a year. It is clear that some metrics were insensitive to on-site visits, remaining triggered. Therefore, metrics need to be periodically checked for sensitivity and trialists should consider how on-site visits interact with their chosen metrics (Are there any activities specifically aimed at dealing with the issue revealed by the triggered metric? If there are, why is this not reflected in post-visit central monitoring reports?)

Trial 2 showed improvements in total trigger scores immediately after the on-site visit for the triggered sites, but a lower proportion are sustained in the long term, and none of the untriggered sites showed improvement immediately after on-site visits. The matrices also show that while Trial 2 monitored 17 metrics, the most across the three trials, the total trigger scores actually reflected the activity of just 10 metrics and two in particular. Again, this suggests the need to assess metrics periodically. It may be that where metrics do not fire it is a sign of good site-performance, or it may mean that the wrong metrics are being used, or that thresholds are inappropriate. Trial teams need to consider this.

Trial 3’s total trigger scores did generally improve after on-site visits. This supports the findings from Trial 1 that scores are sustained over time. However, total trigger scores were volatile at some sites. This is reflected in Figure 4(b), where, unlike in Trial 2, each metric was triggered at some point, even if only once during the study period. This may suggest that the metrics are more sensitive than those in Trial 2. TEMPER trial teams conducted their own risk assessment and chose their own metrics and thresholds, so it is possible that the differences seen here reflect different trial team approaches.^13,24^

The three trials show that, in the majority of cases, total trigger scores decrease immediately after a site visit, although any decrease depends on how high the total trigger score was initially. Sites with untriggered visits, and therefore lower pre-on-site visit total trigger scores, were less likely, or even unable, to lower their total trigger score. In some cases, the next reporting date after a site visit may have been too soon to allow the site to show any change in total trigger score. This can be seen for example in Trial 1 Pair 38 where the decrease in total trigger score for the triggered site only occurred after the post-visit report. Figures 2(b), 3(b) and 4(b) indicate that changes in the number of individual triggered metrics may coincide with visits, show a staggered reduction after the visit or may not seem to change in reference to the visit at all.

This visualisation method allows trialists to look at the pattern(s) over time and between sites within a trial, helping them to consider overall trends rather than get caught up in the detail of each individual trigger. This is useful in learning more about metrics from past trials but can also be used while a trial is ongoing and trial teams are using the metrics as part of monitoring practice.

One limitation is that when TEMPER closed, metric scores stopped being added to the database so were not available for this analysis. Where data was available for a year after the visit, it shows that for Trials 1 and 3 the total trigger score remained the same or decreased over the year in the majority of cases, although for Trial 2 half of the triggered sites saw an increase in total trigger score over the year. While this suggests that the effect of site visits may be lasting in most cases, Figures 2–4 show that total trigger scores may be volatile, increasing above the post-visit report level, before decreasing again. This is particularly apparent for Trial 3.

Figures 2–4 also reveal differences in activity between the three trials. In Trial 3, several graphs of total trigger scores show that at some points in TEMPER, untriggered sites had higher total trigger scores than their paired triggered sites. By contrast, Trial 1 shows a predominantly clear separation between triggered and untriggered metric activity. The metrics chosen for Trial 3 were more volatile, with many triggers firing intermittently. By contrast, Trial two monitored more metrics (17) but also had more metrics that were never triggered, than the other two trials.

The case study is unusual in having paired sites that each received a site visit, allowing two sites to be compared in the graphs and matrices. Those wishing to follow the SWAT 167 protocol are more likely to find themselves creating separate charts for each site.

As this is a case study, we have not used details of the activation and deactivation of metrics and changes to thresholds. However, we know of several examples of such changes, including that three metrics were added to Trial 2 after the first monitoring report was produced,^24^ one of which was triggered. The thresholds of three metrics were also adjusted.^24^ Changes in total trigger score may reflect the introduction of a metric or the change of a threshold rather than an improvement or decline in site performance.

Some post-visit monitoring reports were generated too soon after a site visit for the site visit’s effect to show. Some of the sites were also visited too late in the TEMPER study to be assessed for changes in total trigger score. 12% (9/78) of sites did not have a post-visit report at all, and 51% (40/78) did not have a report a year after the visit (see Table 1). Therefore we do not generalise from our findings to the performance of metrics, focussing on the value of this display.

As presented, the visualisation shows whether a metric has reached a threshold but not the actual metric value. Options to show this should be considered. Other actions could be displayed in the visualisations instead of or as well as on-site visits, e.g. the dissemination of new site guidelines, training activities, or roll out of protocol amendments. Such activities were not recorded in TEMPER. Trialists using this system to review metrics should include such activities, and review their effect.

Using matrices to visualise triggers allows the activity of individual metrics to be seen in context. Substantial information can be encapsulated in one page and can be used in assessing the metrics themselves. If a metric is always triggered across many sites action may be needed to train sites, or the metric threshold may be too sensitive, or a protocol amendment may be needed. Similarly, a metric never reaching its threshold might indicate the trial is running well, or that the threshold needs adjusting. Seeing these patterns in activity allows trial staff to get an overall picture and decide whether they wish to investigate.

Systematic collection of data on metrics allows future investigation of risk-based monitoring strategies. Researchers can conduct studies across multiple trials to look at the impact of certain types of activity on trial metrics, assessing validity of individual metrics, and considering questions about bespoke metrics versus standardised metrics like Whitham’s.^20^ In Box 1 we presented a number of questions raised by a lack of systematically reported real-world experience. By collecting data on trial metrics and examining it using the methods detailed in this paper we can form the basis for answering these questions. For example, finding triggers that do not fire on any trials would point to a need to investigate thresholds and/or look at a different or smaller set of metrics; looking at triggers from many trials across time would give us a better understanding of whether and how long it takes for trial activities to impact on trial metrics and allow us to optimise how frequently triggers should be assessed. This research could in turn lead to the provision of evidence-based guidance on the use of metrics in monitoring. In the meantime, the outlined method will give trialists a tool to assess trial sites and their trial triggers.

### Conclusion

We have shown a visualisation to assess metrics that supports the implementation of risk-based monitoring. The systematic and standardised collection of central monitoring data can:

• provide a simple tool to aid in assessing sites for potential corrective action;
• give staff the ability to see the influence of each metric on total trigger scores and place that influence in context;
• aid the evaluation of metrics and their thresholds; and
• allow the development of a picture of which metrics are most useful, of appropriate thresholds, and of the effectiveness of particular types of monitoring activity.

Our proposed system seeks to standardise the gathering of central monitoring data while allowing trials flexibility in their choice of metrics. We wish to encourage trialists to join in collecting data using our SWAT 167 protocol.^23^ This will allow the clinical trial community to start sharing our monitoring data, and to start working together to establish best practice in Risk-Based Monitoring.

## Supplemental Material

Supplemental Material - Monitoring metrics over time: Why clinical trialists need to systematically collect site performance metrics Supplementary Material for Monitoring metrics over time: Why clinical trialists need to systematically collect site performance metrics by Victoria Yorke-Edwards, Carlos Diaz-Montana, Macey L Murray, Matthew R Sydes, and Sharon B Love in Research Methods in Medicine & Health Sciences.

Supplemental Material - Monitoring metrics over time: Why clinical trialists need to systematically collect site performance metrics Supplementary Material for Monitoring metrics over time: Why clinical trialists need to systematically collect site performance metrics by Victoria Yorke-Edwards, Carlos Diaz-Montana, Macey L Murray, Matthew R Sydes, and Sharon B Love in Research Methods in Medicine & Health Sciences.

## Appendix

### Abbreviations

EMA: European Medicines Agency
FDA: United States Food and Drug Administration
RBM: risk-based monitoring
RCT: randomised controlled trial
SWAT: Study Within A Trial
