# Supplementary material for: Monitoring metrics over time: Why clinical trialists need to systematically collect site performance metrics
Source: Res Methods Med Health Sci. 2022 Dec 21;4(4):124–35. doi: 10.1177/26320843221147855 (PMC7615148; doi:10.1177/26320843221147855)
Supplement: Supplemental Material - Monitoring metrics over time: Why clinical trialists need to systematically collect site performance metrics [file sj-pdf-1-rmm-10.1177_26320843221147855.pdf]

Supplementary Appendix 1

Brief description of dataset

For each TEMPER trial the dataset contains:

- a description of each metric, with;
- details of its set threshold and;
- whether it had to be manually entered or was automatically checked using data from the trial database.

The dataset also contains details of each central monitoring report, with:

- the date of the report;
- the site identifier;
- metric identifier;
- whether the threshold had been met;
- whether the trigger had therefore fired (for metrics where there is a time aspect, e.g. a requirement for a threshold to have been met over two or more reports before firing), and;
- a trigger score (the score given to that metric relating to whether that metric had fired, and the weighting given to that metric by the trial team).

Alongside central monitoring details, the TEMPER dataset also contains data on on-site monitoring visits, including:

- Dates;
- whether the on-site visit was a 'triggered' one (the site had high trigger scores that triggered a visit), or an 'untriggered' one (the site had a low trigger score but had been visited to act as a comparator to a paired high score site), and;
- pair identifier to indicate which two sites were being compared.
