# Supplementary material for: Monitoring metrics over time: Why clinical trialists need to systematically collect site performance metrics
Source: Res Methods Med Health Sci. 2022 Dec 21;4(4):124–35. doi: 10.1177/26320843221147855 (PMC7615148; doi:10.1177/26320843221147855)
Supplement: Supplemental Material - Monitoring metrics over time: Why clinical trialists need to systematically collect site performance metrics [file sj-pdf-2-rmm-10.1177_26320843221147855.pdf]

Metric Definitions by Trial

Trial 1 Metrics

Metric ID	Metric Definition
1	General concern from protocol deviation log
2	Drug dose greater than expected dose
3	Number of missing values of a specific field greater than a given percentage of the total of corresponding forms
4	Number of patients with SAEs is greater than expected
5	Sites who have recruited patients greater than a set target
6	Number of queried fields greater than a given percentage of the total number of available fields
7	Queried fields outstanding for more than a specified time greater than a given percentage of the total of queried fields
8	CRF Return rate less than a given value
9	Enrolment of ineligible patient (date of surgery)
10	Enrolment of ineligible patient (date of bloods)
11	Enrolment of ineligible patient (date of scan)

Trial 2 Metrics

Metric ID	Metric Definition
12	General concern following Trial Management Group meetings
13	Number of patients with SAEs is less than expected
14	Important safety test missed
15	Medication is not administered when a particular test result is below a specified value
16	Sites who have recruited more patients than a set target
17	Number of queried fields greater than a given percentage of the total number of available fields
18	Queried fields outstanding for more than a specified time greater than a given percentage of the total of queried fields
19	CRF Return rate less than a given value
20	Drug (1) dose greater than expected dose
21	Drug (2) dose greater than expected dose
22	Drug (3) dose greater than expected dose
23	Drug (4) dose greater than expected dose
24	Drug (4) given when data indicate it should have been withheld
25	Drug (3) not given at correct dose in Feasibility study centres
36	Drug (5) dose greater than expected dose
37	Number of withdrawn patients more than a given percentage of the total number of patients
38	Drug not given at correct dose

Trial 3 Metrics

Metric ID	Trigger Definition
26	Consent forms return rate less than a given value
27	If death reported, has the relevant form been sent?
28	If progression reported, has the relevant Progression CRF been sent?
29	CRF Return rate less than a given value
30	More than a specified number of patients with last form received more than a given time
31	Number of queried fields greater than a given percentage of the total of available fields
32	Queried fields outstanding for more than a specified time greater than a given percentage of the total of queried fields
33	Number of patients with SAEs is less than expected
34	Number of patients with SAEs is greater than expected
35	General concern following Trial Management Team review
